# Supplementary material for: Exercise as a therapy for sickle cell-associated musculoskeletal pain among children: Healthcare professionals’ perspectives
Source: PLoS One. 2025 Dec 12;20(12):e0327183. doi: 10.1371/journal.pone.0327183 (PMC12700392; doi:10.1371/journal.pone.0327183)
Supplement: S1 File — (DOCX) [file pone.0327183.s001.docx]

**Semi-structured interview guide: Exercise as a therapy for sickle cell associated musculoskeletal pain among children: Healthcare professionals’ perspectives**

Good morning/afternoon, and thank you for taking the time to meet with me today. I am Britney Pinamang Gyampo, a student from the Physiotherapy and Sports Science Department at Kwame Nkrumah University of Science and Technology. I’m here to discuss your views on the role of exercise in managing musculoskeletal (MSK) pain in children with sickle cell disease (SCD).

The interview will take approximately 1 to 1.5 hours, and with your permission, it will be audio recorded to ensure accuracy in capturing your insights. While I have some specific questions prepared, please feel free to share any additional thoughts or experiences you feel are relevant.

To protect your privacy, I kindly ask that you refrain from mentioning your name or any identifying information during the interview. Additionally, please try to speak clearly and at a comfortable pace so that your responses are recorded accurately.

Before we begin, do you have any questions or concerns?

***PART A: Demographics***

1. Gender
2. Experience with Sickle Cell Disease: How long have you worked with patients who have SCD, particularly children?

***PART B: Healthcare professionals' perspective***

3. What is your understanding of the role of exercise in managing joint pain among children with SCD?

4. In your experience, how does exercise prevent joint pain or other complications in children with SCD?

5. What types of exercises do you recommend for children with SCD? How do you decide the intensity and duration of these activities?

6. What barriers do you think prevent children with SCD from participating in exercise programs?

7. Kindly share factors you believe can facilitate the success of exercise programs for these children. How can healthcare professionals help overcome these barriers?

8. What improvements do you think could be made in the use of exercise as a management strategy for SCD-associated joint pain in children?

9. What resources or training do healthcare providers need to better implement exercise-based interventions for children with SCD?

**10.** Is there anything else you would like to add regarding the use of exercise in managing MSK pain in children with SCD?

Thank you
