# Supplementary material for: Exercise as a therapy for sickle cell-associated musculoskeletal pain among children: Healthcare professionals’ perspectives
Source: PLoS One. 2025 Dec 12;20(12):e0327183. doi: 10.1371/journal.pone.0327183 (PMC12700392; doi:10.1371/journal.pone.0327183)
Supplement: Attachment — Submitted filename: RESPONSE TO REVIEWERS (Plos one).docx [file pone.0327183.s003.docx]

**Response to Reviewer’s comments**

Dear Editor,

We genuinely appreciate the reviewer’s valuable feedback on improving the clarity and specificity of the manuscript. We recognize the importance of refining certain aspects to strengthen the overall quality of the study. Please find our detailed responses below for each comment.

**Reviewer #1:**

**Comment:** Please provide more details about the pediatricians and nurses who took part in the interviews. Table 1 provides data on the years of clinical experience, but it is unclear how much of that clinical experience included participating in the care of children with SCD.

**Response**: We thank the reviewer for this important comment. We have now clarified in the manuscript that “All nineteen (19) pediatric nurses and pediatricians who participated in the interviews were directly involved in the treatment of children with SCD. As these hospitals run dedicated SCD clinics weekly, the participants' clinical experience, as reported in Table 1, includes regular and active participation in the care of children with SCD.” Highlighted in **RED on page 5**.

**Comment:** Further, the context of care is important to the interpretation of these results. I assume these healthcare providers were providing inpatient care for acute vaso-occlusive pain, rather than addressing the management of chronic pain, improving physical activity, or attempting to reduce the frequency of acute pain in an outpatient setting.

**Response:** We thank the reviewer. The reviewer's assumption is understandable; however, our study was specifically designed to explore perspectives on exercise, not in the context of acute inpatient pain management, but as a therapeutic strategy for managing persistent musculoskeletal pain and preventing pain crises in the outpatient setting. We intended to investigate a gap in the literature, moving beyond the paradigm of purely pharmacological pain management during crises to explore how pediatricians and pediatric nurses view a proactive, non-pharmacological intervention like exercise for improving long-term health outcomes.

**Comment**: The introduction and discussion with their associated references also need to be clear about these contexts, as the role of exercise in speeding improvement in the resolution of acute pain may be quite different from its role in improving cardiovascular health, improving physical activity, or moderating the effects of chronic pain.

**Response:** We thank the reviewer for this critically important observation. We agree with the reviewer that the distinct roles of exercise in an acute pain context versus a chronic health context are fundamentally different. We have addressed these in the manuscript to reflect the chronic musculoskeletal pain and long-term morbidity in children with SCD, separating it from the acute vaso-occlusive crisis model.

**Reviewer #2:**

**Comment**: Clarity of writing: Some sentences are long and could be streamlined for readability. A thorough language edit would improve flow.

**Response**: We thank the reviewer for this feedback. We agree that enhancing the clarity and readability of the manuscript is important. We have performed a thorough language edit throughout the manuscript, focusing on streamlining long sentences and improving overall flow.

**Comment:** Discussion depth: The link between identified barriers (e.g., kinesiophobia, family concerns) and proposed interventions could be expanded with more practical recommendations.

**Response:** These have been revised to read as “A key psychological barrier is kinesiophobia, the fear of movement due to pain [18]. In SCD, where pain is unpredictable and severe, this fear is particularly understandable. Children may develop avoidance behaviors, which can be unintentionally reinforced by caregivers and clinicians who prioritize safety and pain prevention [19]. This cycle of fear and inactivity paradoxically worsens long-term outcomes by promoting musculoskeletal deconditioning. To break this cycle, clinicians can adopt several practical strategies. A foundational approach is to structure activity program pacing, co-creating an activity plan with the child that begins with short, manageable sessions of low-impact exercise (e.g., 5-10 minutes of walking) and gradually increases in a patient-directed manner. Using an activity diary can provide visual proof of progress and help desensitize the fear that activity always leads to pain. For more complex cases, a proactive referral to a physiotherapist specializing in pediatric chronic pain can facilitate a tailored, graded exposure program, while a clinical health psychologist can address the underlying challenging thoughts using techniques like cognitive behavioral therapy”. Highlighted in **RED on page 15.**

**Comment:** Limitations: The authors acknowledge the limited scope (two hospitals, only nurses and pediatricians). It would be useful to suggest explicitly how including physiotherapists, hematologists, and families in future studies could enrich findings.

**Response**: We have revised the limitation section to read as “The findings are based on data from two hospitals and the perspectives of pediatric nurses and physicians only, which may limit the transferability of the results. Future research should actively include the voices of other critical stakeholders to enrich the findings. For instance, physiotherapists could provide essential expertise on practical exercise prescription and graded movement strategies. Hematologists could offer insights into integrating physical activity with complex medical regimens. Most crucially, including patients with SCD and their families is fundamental to ensuring that any future exercise interventions are feasible, acceptable, and aligned with their lived experiences and priorities”. **Highlighted in RED on page 18.**

**Comment:** Implications for practice: The conclusion could more clearly emphasize actionable recommendations (e.g., integrating physiotherapists into clinic days, caregiver education programs).

**Response**: The implication for practice has been revised and **highlighted in RED on page 19. “**Based on our findings, we propose incorporating physiotherapists or exercise specialists into SCD clinic days to provide real-time assessment, counseling, and the development of individualized activity plans. Again, establish formal channels for pediatricians and nurses to collaborate with hematologists and mental health professionals, ensuring physical activity is addressed as a core component of comprehensive SCD care. Implementing these strategies can help transform exercise from a perceived risk into a standard, managed therapy for improving health outcomes in this population.”
