# Supplementary material for: Exercise as a therapy for sickle cell-associated musculoskeletal pain among children: Healthcare professionals’ perspectives
Source: PLoS One. 2025 Dec 12;20(12):e0327183. doi: 10.1371/journal.pone.0327183 (PMC12700392; doi:10.1371/journal.pone.0327183)
Supplement: Attachment — Submitted filename: Response to Reviewer (PLOS ONE ).docx [file pone.0327183.s004.docx]

**Response to Reviewer’s comments**

Dear Editor,

We appreciate the reviewer’s valuable feedback on improving the clarity and specificity of the manuscript. Please find our detailed responses below for each comment.

Reviewer #3: 
**comment:** Add practical exercise examples: Including specific exercises suitable for children with SCD—like low-impact or graded activities—would give clinicians clearer takeaways and link perceptions to real-world practice.

**Response:** We thank the reviewer for this important comment. However, we have added practical exercise examples. Highlighted on page 14 and read as “Such a program, like 'play-based & social activities' (indoor recreational games, light ball play, dancing, and performing daily chores under supervision),

**Comment:** You discuss physiotherapists in the limitations, but acknowledging their role earlier could make the discussion of implementation barriers clearer.

**Response:** We appreciate the reviewer’s insightful observation. The current study specifically explored healthcare professionals' (pediatric nurses’ and pediatricians’) perspectives on exercise as a therapy for sickle cell–associated musculoskeletal pain among children. As such, the inclusion of physiotherapists was beyond the original scope and participant design of the study. The intention was to capture how frontline medical and nursing professionals perceive, recommend, or integrate exercise within pediatric sickle cell care, given their frequent patient contact in clinical and ward settings. Participants acknowledged that their availability will enhance the implementation of the exercise in the management of MSK in SCD. However, we fully acknowledge that physiotherapists play an important role in exercise prescription, movement retraining, and graded activity planning for children with sickle cell disease. Their perspectives would indeed deepen understanding of implementation feasibility and enhance interdisciplinary insight. In line with this, we have clarified their potential contribution earlier in the discussion to strengthen the connection between professional collaboration and implementation barriers. We have also noted the need for future studies to include physiotherapists and other rehabilitation professionals to provide a more holistic view of exercise-based interventions for this population.

**Comment**: Check flow and readability: A few of the longer sentences could be tightened for smoother reading throughout the manuscript.

**Response**: Thank you for the observation. The longer sentences have been reviewed and revised throughout the manuscript to improve flow, clarity, and readability.

**Comment:** Clean up references: There are a few repeated or inconsistently formatted references; standardizing them would improve overall presentation.
**Response:** Thank you for the observation. The reference list has been thoroughly reviewed, and repeated or inconsistently formatted entries have been corrected.
